# Modeling the stochastic behavior of lupus

**DOI:** 10.1186/ar4622

**Published:** 2014-09-18

**Authors:** Derry C Roopenian, Elisabeth B Adkins, Giljun Park, Herbert C Morse, Gregory W Carter

**Affiliations:** 1 The Jackson Laboratory, Bar Harbor, ME, USA; 2 Sackler School of Graduate Biomedical Sciences, Tufts University, Boston, MA, USA; 3 National Institute of Allergy and Infectious Diseases, Rockville, MD, USA

## Background

### Looking forward to preclinical risk assessment and early interventions

A major aspiration of clinical medicine is early diagnosis and interventions that interrupt autoimmune diseases in its earliest stages, before they become increasingly difficult and dangerous to treat. Definition of the relative contributions of three critical components at the earliest phases of disease is required to realize this goal: 1) genes; 2) environment; and 3) chance. Lupus, like all common human disorders, results from the interplay of these components. Currently >35 allelically variant genes, many of which are shared with other autoimmune disorders, are reported to contribute to the pathogenesis of lupus. The roadmap for genetic risk assessment is relatively straightforward. Even given that present studies are confounded by the failure of defined genetic variation to fully account for disease heritability, definition of the genetic components can be generated digitally through deep genome sequencing, genome wide association (GWA) studies and family linkage studies. However, thinking forward to the best-case scenario in which all heritability is explained, there will still be a substantial gap. This gap is commonly attributed to unknowns casually referred to as environmental factors and chance events. The importance of these extragenetic unknowns is underscored by studies of monozygotic twins, which have typically revealed >70% discordance for common autoimmune syndromes, including lupus. While epigenetic mechanisms may eventually explain some of this discordance, nebulously defined environmental factors and chance will continue to cloud genome-based risk prognostications and limit the potential for identifying individuals with sufficient genetic evidence to justify early interventions.

### What are contributions of environment?

The identification of relevant environmental factors is in its infancy. Lupus flares caused by sunburn and drug-induced lupus-like syndromes stand out as well described environmental triggers, but the larger question of environmental exposure history, including prior viral infections, gut microbiome composition, chemical exposures, and what have you, that promote lupus pathogenesis will only be incrementally solved. Extracting the contributions of environmental factors would be made more tractable by first understanding the extent to which chance confounds the ability to derive cause-effect relationships between environment and disease.

### What is the contribution of chance?

That which cannot be attributed to environmental factors would logically fall into the nebulous category of chance, termed more scientifically as stochastic behavior. This indeterminate parameter is increasingly recognized to be an integral component of all physical and biological systems. The prototype is Brownian motion of a particle traveling through a solution and colliding with molecules in a random manner. While random and unpredictable at the inception, stochastic behavior become biologically relevant if the "collision" initiates a chain of molecular and/or cellular processes that evolve into measurable "deterministic" behaviors. Thus, the chance engagement of molecule A with molecule B within a critical cell type cell or the chance cognate engagement of a critical cell type (such as a naïve CD4^+ ^T cell) with an antigen presenting cell (APC) could trigger the development of lupus.

### The intrinsic variability of inbred strains of laboratory mice provides a means to understand the impact of stochastic behavior on disease

Investigation into the potential contributions of stochastic factors in complex disease processes is not practical in humans. Inbred strains of laboratory mice are the mammalian organism best suited for the task. Missing heritability as a confounding factor is negligible in highly inbred laboratory mice of the same inbred strain because each mouse is an identical twin. Environmental variation that cannot be readily controlled in humans can be minimized in mice by consistent husbandry in a stable laboratory colony.

### The BXSB.*Yaa *model of lupus

BXSB male mice carrying the *Yaa *mutation spontaneously develop a systemic autoimmune disease with multiple similarities to severe forms of human lupus. A duplicated copy of Toll-like receptor 7 (*Tlr7*) caused by the *Yaa *mutation is the primary genetic cause of this lethal lupus-like disease. The consequence of the *Tlr7 *duplication - realized only in *Yaa *males - is the potent evocation of the IFN1 response starting at a remarkably young age and the development of follicular T cells (T_FH_) that secrete IL21 and drive massive germinal center B cell and plasmablast expansions. This results in high levels of autoantibodies to RNA, DNA and other self-antigens, formation of nucleic acid containing immune complexes and lethal immune complex-mediated glomerulonephritis that lead to premature deaths.

## Methods and results

### Robust variation in the autoimmune disease outcomes is apparent in individual BXSB.*Yaa *with uniform genetics and husbandry environment

BXSB.*Yaa *mice have been inbred for at least 100 generations, 50 of which were performed in our colony. At least to the depth of genetic analysis performed to date, our BXSB.*Yaa *colony is genetically fixed. Moreover, continuous microbial monitoring has not revealed any changes over the last 20 years. Thus as a first approximation the environment in our BXSB.*Yaa *research colony is notably stable.

**Figure 1:**
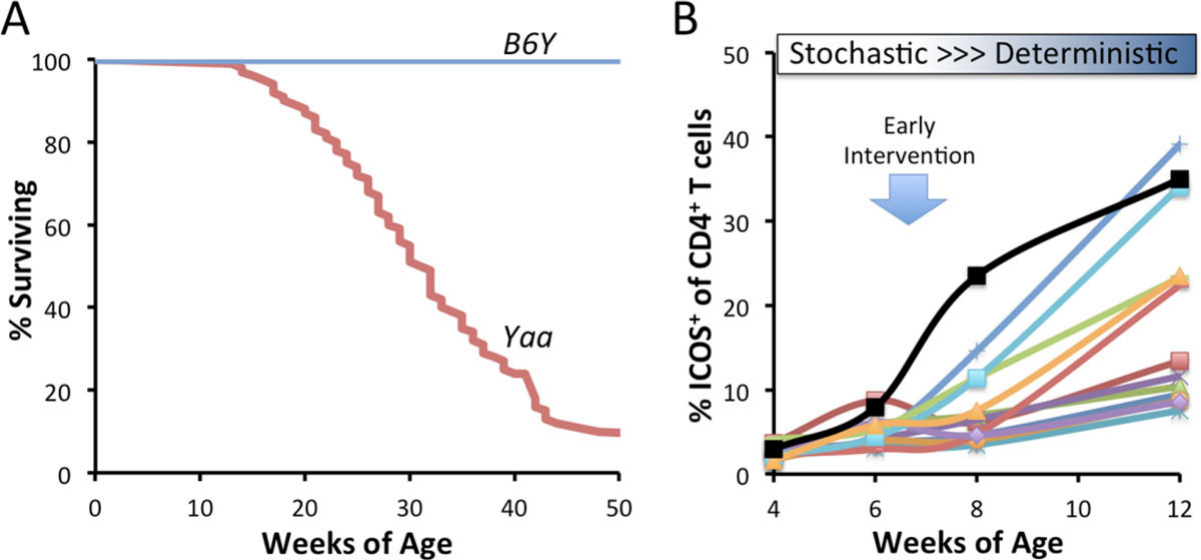
**A, Considerable individual variation in the survival of BXSB**. *Yaa *male mice, n = 93. Long term survival of 26 males carrying the C57BL/6 Y chromosome (*B6Y*) demonstrate the dependency of the autoimmune disease on *Yaa*. **B**, Longitudinal phenotyping of a cohort of BXSB.*Yaa *mice for ICOS^+ ^CD4^+ ^T cells. Each line represents data from FACS analysis of blood white blood cells from individual mice. Transition from stochastic to deterministic occurs at 6-8 weeks of age. Therapies applied at this point are predicted to have the potential of preventing this transition.

However, we repeatedly observe substantial variation in the timing and severity of autoimmune disease among individual, highly inbred BXSB.*Yaa *mice in well-powered studies measuring overall mouse survival (example Figure 1A). This variation is not consistent with genetic drift and/or fitness selection for healthier breeders. Substantial disease variation determined here by outcomes (timing of survival) is a durable feature of inbred BXSB.*Yaa *mice.

### Robust variation in longitudinal expression of T_FH _in blood is observed in BXSB.*Yaa *mice at early stages of disease

Variation in the survival times of BXSB.*Yaa *would predict that biomarkers of mechanistically important cellular processes would vary similarly. Variation in such biomarkers at early stages of disease would be of most value because they may identify targets for early therapeutic interventions. T_FH _are critical drivers of the BXSB.*Yaa *disease. Preliminary longitudinal studies investigating the expression of circulating ICOS^+ ^CD4^+ ^T cells show remarkable individual variation in the frequencies of these cells by at least 8 weeks of age (Figure 1B). While such results are not yet linked to the timing of survival of the mice or correlated with other important biomarkers of disease, it is reasonable to surmise that stochastic events acting within weeks of birth bifurcate into important deterministic processes (activation and expansions of T_FH _in this case) that may be have long term consequences.

## Conclusions

### Much can be learned by embracing the concept of stochastic behavior

The principles described here are unlikely to be restricted to BXSB.*Yaa *mice. Virtually all genetically homogenous models of disease demonstrate a range of variation in disease timing and severity. It is therefore likely that stochastic principles broadly underlie the individual variations commonly observed in inbred mouse models of disease. However, such variation is usually disregarded. Conventional experimental studies are usually designed to override "stochastic noise" by attempts to size comparator cohorts with sufficient discriminative power to override individual variability. When significance in cohort comparisons is not achieved, the null hypothesis is invoked. The individual variability within genetically matched cohorts is an untapped source of biological information that can inform causal mechanisms of disease.

### Extrapolation to disease prognosis and early interventions in humans

Variation in the severity and presentation among individuals diagnosed with a human autoimmune disease is the rule rather than the exception. While genetic risk and environment factors are certain to play important parts, the considerable phenotypic variation described above underscores the potential for stochastic behaviors to contribute significantly to disease variation. Genetically and environmentally fixed BXSB.*Yaa *mice exhibit patterns that are consistent with stochastic events giving rise to key deterministic events at the inception of autoimmune disease. By extrapolation, therapeutic interventions designed to abrupt such early fate decisions may head off lupus at its inception and have enduring effects on overall disease pathogenesis (Figure 1B).

